# Performance of a Trocar Sleeve Adapter for Faster Silicone Oil Extraction in Real-World Vitreoretinal Surgery

**DOI:** 10.3390/jcm14176052

**Published:** 2025-08-27

**Authors:** Philip Wakili, Colya N. Englisch, Peter Szurman, Clara E. Englisch, Clemens N. Rudolph, Núria Pérez Guerra, Anna Theresa Fröhlich, Boris V. Stanzel, Karl T. Boden

**Affiliations:** 1 Eye Clinic Sulzbach, Knappschaft Hospitals Saar, 66280 Sulzbach/Saar, Germany; 2 Klaus Heimann Eye Research Institute (KHERI), 66280 Sulzbach/Saar, Germany

**Keywords:** vitreoretinal surgery, vitrectomy, silicone oil, heavy silicone oil, low viscosity, high viscosity, high flow cannula, removal, 23G trocar, tubeless siphoning

## Abstract

**Objectives**: To assess the performance of a high-flow viscous-fluid-extraction cannula as a 23G trocar sleeve adapter (1362.VFE2, DORC, Zuidland, the Netherlands) for removing regular and heavy silicone oil (SO) in real-world vitreoretinal surgery. **Methods**: In this retrospective study, 90 eyes undergoing SO removal were analyzed. The 23G trocar sleeve adapter was evaluated in 30 eyes for regular SO (DORC Silicone 5000) and 30 eyes for heavy SO (Densiron^®^ 68). The latter were compared with a standard disposable heavy-SO-extraction cannula (1272.HSE06, DORC) in another 30 eyes. **Results**: Removal of DORC Silicone 5000 using the trocar sleeve adapter required 332.0 ± 122.8 s. To extract Densiron^®^ 68, the adapter was significantly faster than the standard heavy-SO cannula (162.6 ± 85.6 s vs. 619.3 ± 128.8 s; *p* < 0.0001). **Conclusions**: The 23G trocar sleeve adapter efficiently extracts both regular and heavy SO, reducing the time required to extract Densiron^®^ 68 by four-fold compared with a dedicated heavy-SO cannula. Its use can markedly shorten surgical time in real-world vitreoretinal procedures.

## 1. Introduction

Silicone oils (SOs) are commonly used as long-term endotamponades in vitreoretinal surgery. However, low-viscosity SOs are prone to vision-impairing emulsification [1,2,3], whereas high-viscosity SOs offer greater stability, but require significantly longer injection and extraction times [4]. In our experience, filling an entire eye with standard equipment and high-viscosity SO requires 13 min on average. Prolonged injection and extraction times may contribute to increased infection risk, higher surgery costs [5], and greater risk for light-induced retinal damage. This challenge is amplified by the ongoing shift toward microinvasive vitreoretinal surgery, which involves smaller instrument diameters. According to the Hagen–Poiseuille law, flow increases with the pressure gradient and the fourth power of the radius but decreases with system length and fluid viscosity. In standard setups, the narrowest segment for SO flow is the trocar, as both the cannula’s outer and, consequently, inner radius introduced into it are limited by its small inner diameter. The trocar sleeve adapter which is investigated herein fits directly over the trocar head, eliminating the need for a conventional cannula and increasing the inner diameter at this critical point, boosting the flow rate. Our recent study reported 10-fold faster SO injection in a laboratory setting [6].

To date, the clinical application of trocar sleeve adapters for SO extraction is insufficiently investigated. Our aim was to retrospectively evaluate the performance of such a trocar adapter in explanting SOs in real-world vitreoretinal surgery.

## 2. Methods

### 2.1. Materials

**Figure 1:**
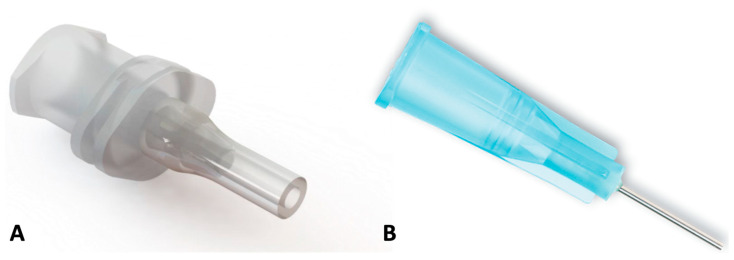
Trocar sleeve adapter (**A**) and standard heavy silicone oil extraction cannula (**B**). Image provided by DORC International.

For the intervention group, we used a high-flow viscous-fluid-extraction accessory cannula as a trocar sleeve adapter (1362.VFE2, DORC (Dutch Ophthalmic Research Centre, Zuidland, The Netherlands) and a disposable heavy-SO extraction cannula for the control group (1272.HSE06, DORC, Zuidland, the Netherlands) (Figure 1). In both groups, 23G AVETA trocar systems (DORC, Zuidland, The Netherlands) were employed. Low-viscosity, heavy Densiron^®^ 68 SO (1400 mPas, Fluoron GmbH, Ulm, Germany) and high-viscosity, regular DORC Sil-5000-S SYRINGE SO (5000–5900 mPas, DORC, Zuidland, The Netherlands) were used.

### 2.2. Study Design

This single-center retrospective study adhered to the Declaration of Helsinki and was approved by the local Institutional Review Board (Ethikkommission bei der Ärztekammer des Saarlandes, 243/14). Written informed consent was obtained from all participants. Inclusion criteria were removal of Densiron^®^ 68 or DORC Silicone 5000 at Eye Clinic Sulzbach between April 2024 and April 2025; exclusion criteria were lack of consent, age of <18 years, and pregnancy.

### 2.3. Surgery

Indication for surgery was SO removal. The standard surgical setup included the placement of three trocars: one for SO aspiration, one for balanced salt solution (BSS) infusion, and one for light pipe-based endoillumination.

#### 2.3.1. Trocar Sleeve Adapter Technique

**Figure 2:**
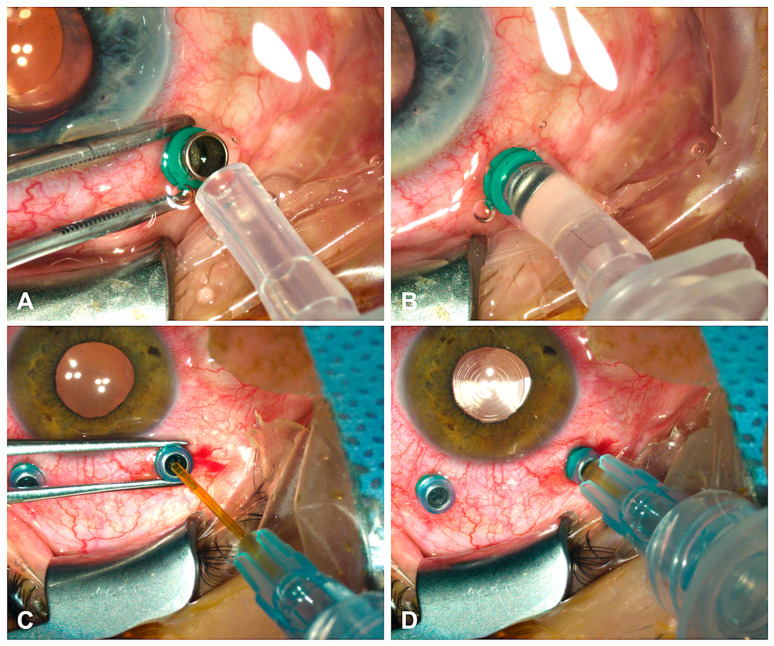
Application of the trocar sleeve adapter to the 23G trocar head (**A**,**B**), and insertion of the standard heavy silicone oil extraction cannula into the 23G trocar head (**C**,**D**).

For SO extraction, the BSS infusion pressure was set to 45 mmHg. After removing the valve cap from the 23G trocar, the SO aspiration tube of the EVA system was connected to the trocar sleeve adapter, which was mounted directly over the trocar head (Figure 2A,B). SO was aspirated under wide-angle visualization (RESIGHT, Carl Zeiss Meditec, Jena, Germany) using a negative pressure of −660 mmHg. Air bubbles were removed using a flute needle. The intraocular pressure (IOP) was then adjusted to 15–20 mmHg using a Schiötz tonometer (Geuder AG, Heidelberg, Germany). After surgery, sclerotomies were sutured with 8-0 Vicryl (Ethicon Inc., Raritan, NJ, USA).

#### 2.3.2. Standard Cannula Technique for Extracting Heavy Silicone Oil

To extract Densiron^®^ 68 using the heavy-SO cannula, removal of the valve cap was unnecessary. The cannula was inserted through the 23G trocar and connected to the EVA system (Figure 2C,D). BSS was infused at 45 mmHg, and SO was aspirated under a negative pressure of −660 mmHg. After complete SO removal, the procedures described above were carried out.

### 2.4. Time Measurement

Procedure time was recorded from the initiation of foot-pedal-controlled aspiration until the final SO droplet was removed from the vitreous cavity. The time required for IOP adjustment and/or silicone emulsification washout was excluded.

### 2.5. Assessment

The primary outcome was the time required for SO extraction. Secondary outcomes included corrected distance visual acuity (CDVA), measured preoperatively and at three months postoperatively, and IOP, measured preoperatively and at six weeks postoperatively. Intraoperative and postoperative complications were documented. Postoperative SO status was evaluated during a routine control slit-lamp examination.

### 2.6. Statistical Analysis

Data are presented as mean ± standard deviation (SD). Normality was tested using the Shapiro–Wilk test. Demographic comparisons were made using the Kruskal–Wallis test for continuous variables or Fisher’s exact test for categorical variables. Outcome parameters were analyzed using the Kruskal–Wallis test, followed by Dunn’s multiple comparison test. The relationships between extraction duration and age, axial length, vitreous volume, and preoperative IOP were investigated using Spearman correlation. Two-sided *p*-values < 0.05 were considered statistically significant. Analyses were performed using GraphPad Prism software (version 10.2.2, GraphPad Software Inc., San Diego, CA, USA).

## 3. Results

### 3.1. Demographics

A total of 90 eyes from 90 patients undergoing SO extraction were included. In 30 eyes, DORC Silicone 5000 was extracted using the trocar sleeve adapter. In 60 eyes, Densiron^®^ 68 was extracted using either a standard heavy-SO cannula (*n* = 30) or the 23G trocar sleeve adapter (*n* = 30). No cases involved DORC Silicone 5000 extraction using the standard heavy-SO cannula, and given that alternative extraction methods for regular SOs are no longer used by our clinic, a real-world control group for this cohort was not established. Table 1 shows the baseline characteristics of all cohorts, as well as the time course of CDVA and IOP. No significant differences were observed between the three groups regarding age, sex, main diagnosis, axial length, vitreous volume calculated using the VIVEX formula [7], or CDVA and IOP.

### 3.2. Safety

**Figure 3:**
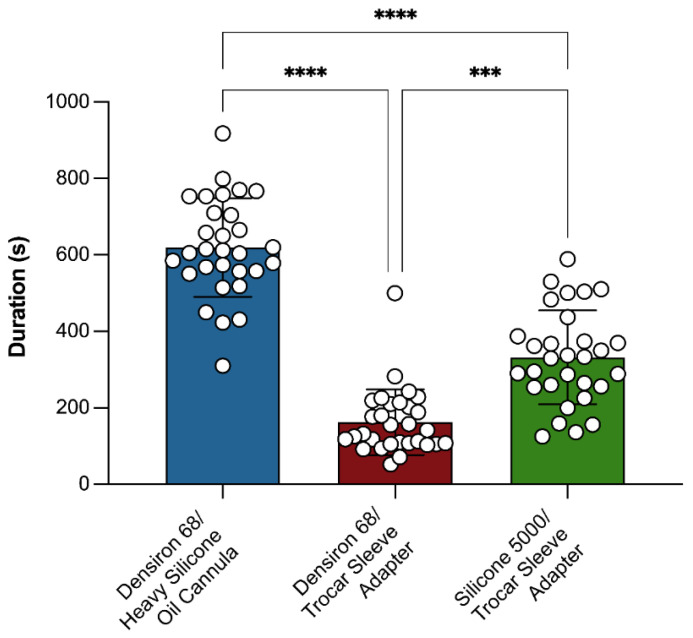
Duration of silicone oil (SO) extraction. Densiron^®^ 68 was extracted using the 23G trocar sleeve adapter (red) or the standard heavy-SO cannula (blue). DORC Silicone 5000 was extracted using the 23G trocar sleeve adapter (green). Bar graphs represent mean ± SD; individual values are shown as white symbols. *** indicates *p* < 0.001, **** *p* < 0.0001.

In the Densiron^®^ 68 cohort, the trocar sleeve adapter had to be re-sleeved over the trocar once. After reattachment, extraction proceeded, but resulted in the longest observed duration (500 s) of this group (Figure 3). No major complications occurred in any of the cohorts.

### 3.3. Silicone Oil Extraction Duration

Extraction of high-viscosity DORC Silicone 5000 using the trocar sleeve adapter required 332.0 ± 122.8 s. Low-viscosity, heavy Densiron^®^ 68 was removed four times faster using the 23G trocar sleeve adapter (162.6 ± 85.6 s) than the heavy-SO-extraction cannula (619.3 ± 128.8 s; *p* < 0.0001) (Figure 3). Of note, extraction duration, overall, did not correlate with age at surgery (r = 0.01, *p* = 0.9) or preoperative IOP (r = 0.08, *p* = 0.5) but with axial length (r = 0.2, *p* = 0.04) and vitreous volume (r = 0.2, *p* = 0.04). In all eyes, no relevant amounts of SO remained after extraction, as determined during slit-lamp examination.

## 4. Discussion

This study shows that the trocar sleeve adapter is a high-performance tool for the extraction of regular SOs. Importantly, it also demonstrates that heavy SOs such as Densiron^®^ 68 can be extracted much faster using the trocar sleeve adapter than a conventional heavy-SO-extraction cannula. Notably, age and preoperative IOP showed no correlation with extraction duration, whereas axial length and vitreous volume showed correlations. Our findings carry both clinical and economic significance. Heavy-SO removal surgery typically requires between 30 and 60 min, even for experienced surgeons. The average time savings of 7.6 min observed in this study may contribute to reducing infection risk and light-induced retinal damage, while also shortening the surgical time and lowering associated operating-room costs, as previously discussed [6,8,9]. Our results also align with broader surgical trends aimed at minimizing instrument size to enhance precision in vitreoretinal procedures.

Originally developed for the efficient removal of regular SOs such as DORC Silicone 5000, the trocar sleeve adapter is mounted on the trocar and aspirates through the trocar’s internal lumen. By contrast, the conventional heavy-SO-extraction cannula is inserted directly into the vitreous cavity via a metal tip ~10 mm in length. This design difference led to the assumption that the trocar sleeve adapter was unsuitable for heavy-SO extraction, primarily owing to the lack of direct intravitreal access. Our clinical findings challenge that assumption. Under continuous negative pressure, Densiron^®^ 68 removal was both safe and complete using the trocar sleeve adapter. In the case of DORC Silicone 5000, as the SO volume decreased and the vitreous cavity filled with BSS, the SO floated and was aspirated without interruption. By contrast, Densiron^®^ 68, which is denser than BSS, progressively lost contact with the trocar insertion as the SO volume decreased. Nevertheless, with continuous suction, the viscoelastic flow properties of the heavy Densiron^®^ 68 allowed it to ascend toward the trocar [10]. This phenomenon, termed “tubeless siphoning”, has been thoroughly described by Stappler *et al*. [10]. When Densiron^®^ 68 is aspirated through a narrow aperture, it may behave as a non-Newtonian fluid: as its molecular structure unfolds, energy is stored, allowing for complete removal [10,11,12].

While we observed some disconnections during SO injection in real-world practice, no disconnections occurred during extraction. This can be attributed to the negative pressure within the system, which maintains a secure connection between the trocar and the sleeve adapter. In one case, necessary re-sleeving of the adapter over the trocar was complicated by contact with Densiron^®^ 68, accounting for the observed singular longer extraction time (8.3 min). To prevent such complications, a screw or click mechanism could be implemented to securely fix the adapter to the trocar.

The concept of sleeving a device over a trocar for accelerating SO extraction is not new. For example, Song *et al*. used a modified blood-transfusion system sleeved over a 23G trocar after valve removal [13]. Zhang and Zhang described a similar trocar-sleeving technique using 27G trocars and manually operated 10 mL syringes for SO extraction [14]. Most recently, Hammer *et al*. applied a trocar sleeve adapter from Alcon (Fort Worth, TX, USA) in a laboratory setting. Their findings support the idea that removing the trocar valve and applying a trocar adapter can significantly accelerate SO removal [15]. Although our results align with theirs, several of their concerns regarding trocar sleeve adapters did not reflect our experience. First, they suggested that angulation of the trocar tip could be impaired by the adapter [15]. In our protocol, however, SO removal is performed entirely along the trocar’s axis. Angulation is neither required nor advantageous. Conclusively, this theoretical limitation holds no relevance, and we encountered no maneuverability issues with the adapter. Second, they noted a potential risk of disconnection owing to minor movements [15]. While this concern may apply to SO injection, we found that during real-world extraction, the negative pressure sufficiently stabilized the connection. Lastly, they stated that their extraction sleeve could not be used for SO injection [15]. Our previous study, which used the same trocar sleeve adapter investigated herein, demonstrated the opposite: SO injection was significantly faster than using a standard infusion tube [6]. Furthermore, while Hammer *et al*. proposed that hybrid techniques might be required to efficiently remove high-viscosity SOs [15], we found that the trocar adapter alone is sufficient for complete and rapid extraction of both high-viscosity and heavy SOs. Other strategies to improve SO extraction performance are conceivable, such as using cannulas with large inner diameters, employing a dual-trocar setup [16,17], or introducing luer trocars [15]. However, previous research has shown that a 25G sutureless approach outperforms a 20G approach, which requires suturing, in terms of surgical speed [18].

## 5. Conclusions

In this study, the trocar sleeve adapter consistently reduced intraoperative SO extraction time and facilitated removal without compromising completeness or increasing complication rates, demonstrating feasibility and promising performance. DORC Silicone 5000 extraction using the trocar sleeve adapter was investigated as a proof of concept, while the adapter and a standard cannula were compared for extraction of Densiron^®^ 68. The simple sleeving mechanism of the trocar adapter makes it a practical, efficient, time-saving tool for both heavy and regular SO extraction in modern vitreoretinal surgery. Limitations include the retrospective design, small sample size, absence of a matched DORC Silicone 5000–heavy SO extraction cannula cohort, and lack of random allocation. These factors restrict generalizability, and the findings should be confirmed in larger, prospective, randomized studies with multivariable adjustment.

## Figures and Tables

**Table 1:** Baseline characteristics and longitudinal corrected distance visual acuity (CDVA) and intraocular pressure (IOP) measurements across the three cohorts.

		Densiron 68/ Heavy Silicone Oil Cannula	Densiron 68/ Trocar Sleeve Adapter	Silicone 5000/ Trocar Sleeve Adapter	*p*
**Total (*n*)**		30	30	30	>0.99
**Sex (*n*)**	Male Female	17 13	21 9	17 13	0.5
**Age (Years)**		66 ± 9.7	61 ± 12.7	51 ± 22	0.06
**Eye (*n*)**	Right Left	17 13	16 14	18 12	0.96
**Axial Length (mm)**		24.0 ± 1.9	23.8 ± 2.5	23.4 ± 1.7	0.1
**Vitreous Volume (cm^3^)**		5.7 ± 1.4	5.5 ± 1.6	5.1 ± 1.2	0.1
**Main** **Diagnosis (*n*)**	Rhegmatogenous Retinal Detachment	26	27	25	0.1
	Tractional Retinal Detachment	1	2	5	
	Full-Thickness Macular Hole	3	0	0	
	Trauma	0	1	0	
**Visual Acuity** **(LogMAR)**	Preoperatively Postoperatively	1.0 ± 0.4 0.7 ± 0.5	1.2 ± 0.5 1.0 ± 0.7	1.3 ± 0.7 1.0 ± 0.7	0.5 0.3
**Intraocular Pressure (mmHg)**	Preoperatively Postoperatively	18.0 ± 7.9 16.1 ± 4.4	15.9 ± 5.2 15.1 ± 6.1	16.0 ± 6.2 15.2 ± 4.9	0.8 0.7

## Data Availability

The raw data supporting the conclusions of this article will be made available by the authors on request.
